# Evaluation of Salivary Parameters and Oral Health Status Among Asthmatic and Nonasthmatic Adult Patients Visiting a Tertiary Care Hospital

**DOI:** 10.7759/cureus.5957

**Published:** 2019-10-21

**Authors:** Rukshana Fathima, Rekha Shenoy, Praveen S Jodalli, Laxminarayan Sonde, Imran P Mohammed

**Affiliations:** 1 Public Health Dentistry, Yenepoya Dental College and Hospital, Mangalore, IND

**Keywords:** asthma, dental caries, saliva, salivation, salivary ph, salivary flow rate, salivary calcium

## Abstract

Introduction: Asthma is a chronic inflammatory disorder of the airways characterized by attacks of bronchoconstriction causing shortness of breath, coughing, and rapid breathing. Patients undergoing inhalation therapy are subjected to a higher risk of dental caries due to the reduced saliva secretion, decreased pH value, increased number of cariogenic bacteria caused by inappropriate oral hygiene, and the use of inhaling medicaments with dry powder.

Methods: The descriptive study was carried out among 200 patients aged (18-45 years) visiting Yenepoya Hospital, Mangalore during the period from May 2018 to October 2018 to evaluate salivary parameters and oral health status in asthmatic and nonasthmatic patients. Data were collected using WHO criteria (2013) and samples of unstimulated saliva were collected for evaluation of salivary flow rate, pH, buffering capacity, and calcium. Statistical analysis was done using SPSS version 21.0 software with the level of significance set at p < 0.05.

Results: A decreased mean score of salivary flow rate, pH, and buffering capacity were found in asthmatic patients compared to nonasthmatic patients (p < 0.001). A higher mean decayed, missing, and filled teeth (DMFT) score and an increased dental erosion score were found in asthmatics compared to nonasthmatics (p < 0.05). Asthmatics had higher gingival bleeding score than nonasthmatics.

Conclusion: In order to prevent disease progression in the hard dental tissue and oral cavity of asthmatic patients, it is necessary to apply adequate preventive measures and motivate their continuous application in this caries-risk population.

## Introduction

Asthma is a chronic inflammatory disorder of the airways characterized by attacks of broncho-constriction causing shortness of breath, coughing, chest tightness, and rapid breathing. The severity of asthma can be classified as mild, moderate, or severe. The prevalence of asthma has been increasing since the 1980s across all ages, gender, and racial groups. Asthma currently affects 300 million people worldwide and an additional 100 million people are estimated to get affected by 2025 [1]. Among India’s 1.31 billion people, about 6% of children and 2% of adults have asthma [2]. Asthma treatment has two main parameters: to control, as well as to decrease the airway inflammation, and re-establish the airways. Drugs that attain the first objective are known as anti-inflammatory agents and those that attain the second are known as bronchodilators. Most asthma drugs are inhaled using various forms of inhalers or nebulizers. Inhalers which deliver these medicaments have to be used up to four times a day over a long period [1].

 Saliva which plays a major part in the health of the mouth has various functions in the oral environment like clearing of food debris and bacteria, buffering capacity on tissue-damaging strong bases and acids, and providing saturated solution of calcium needed in remineralization of teeth. The amount of saliva secreted in the oral cavity and its constituents has a role in the incidence of dental caries [3]. Asthmatic patients with prolonged use of β2-agonists are subjected to a higher risk of dental caries due to the reduced saliva secretion, decreased pH value, decreased buffering capacity, increased number of cariogenic bacteria caused by inappropriate sugar consumption, oral hygiene, and the use of inhaling medicaments with dry powder. The powder versions of preventer therapies, e.g. Becotide and Flixotide and relief bronchodilators, e.g. Ventolin and Bricanyl, the mainstays of asthma treatment, are acidic with a pH below 5.5. There may be a fall in the pH of interdental plaque and saliva during the 30 minutes following the use of inhaler medication for asthma. Enamel starts to dissolve below a pH of 5.5. The powder in the puffers can erode the tooth enamel when used regularly [4].

Asthmatic patients may be at a higher risk of advancing various dental diseases like dental caries, periodontal diseases, dental erosion, etc. Dental practitioners or dentists should be apprised of the correlation between asthma and oral health. They should train and counsel their patients to follow preventive measures to intercept adverse effects on hard and soft tissues of the oral cavity. Simple measures can be undertaken to decrease these diseases by referral of the asthmatic patient by the attending physician to the dentist, or educating the patient on the necessary oral hygiene and preventive measures. The primary objective of the study was to compare oral health status and salivary parameters among asthmatic and nonasthmatic adult patients. This study will help to give more emphasis on regular oral health maintenance for those with asthma and for more research into the possible protective ways in limiting oral health problems among asthmatic patients.

## Materials and methods

 Study design, settings, and sample

It was a descriptive cross-sectional study conducted in a tertiary care center in Mangalore. Institutional review board approval was attained from the Institutional Review Board of Yenepoya University and written informed consent was attained from all participants. The estimated sample size = 100 in each group with a level of significance 5% and power 90%. Inclusion criteria for sample recruitment were adult:

1) Asthmatic patients of age 18 years and above using asthmatic medications for at least six months or using an inhaler device for management of asthma, attending Pulmonary Medicine Department, Yenepoya Medical College Hospital. 2) Nonasthmatic patients of age 18 years and above from persons accompanying the patients visiting the Department of Pulmonary Medicine and other departments of Yenepoya Medical College Hospital. Noncooperative patients or patients who do not consent to participate in the study. The patients below 18 years of age were excluded from the analysis.

Study variables

Demographic details and brief medical history of the participants are recorded. The oral health status among asthmatic and nonasthmatic adult patients will be assessed by WHO criteria (2013) [5]. The following information was recorded using the oral health form, general information, extraoral conditions, dentition status (crown, root), periodontal status, loss of attachment, enamel fluorosis, dental erosion, dental trauma, and oral mucosal lesion.

Samples of unstimulated saliva were collected for evaluation of salivary flow rate, pH, buffering capacity, and calcium. Salivary flow rate was measured using precision balance weighing. The salivary pH was directly estimated using the digital pH meter. Salivary buffering capacity was assessed by the Ericsson’s method. Salivary calcium level was estimated using Calcium ChemKit (Agappe Diagnostics Pvt. Ltd., Kochi, India) [6-9].

Statistical analysis

The collected data were entered into Microsoft excel software by the examiner. The entered data were exported to SPSS 22.0 software for statistical analysis. Descriptive statistics are presented in the form of a frequency percentage. Unpaired ‘t’ test was used to compare the mean decayed, missing, and filled teeth (DMFT), mean in salivary parameters like salivary flow rate, pH, buffering capacity, and calcium between asthmatic and nonasthmatic patients. Mann-Whitney U test was used to compare the mean periodontal pockets and mean gingival bleeding.

## Results

Table 1 shows the distribution of the participants by age. Among the 100 asthmatic cases, 28% were there in the age group of 25-30 years and 25% were in the age group 31-35 years. Similarly, among the 100 nonasthmatic cases 33% were in the age group of <25 years and 43% were in the age group of 25-30 years. In both asthmatic and nonasthmatic group, the maximum number of the subjects was in the age group of 25-35 years. The difference was found to be statistically significant (p = 0.002).

**Table 1:** Age distribution of the participants. *x*^2 ^= 16.832;  p = 0.002 hs hs: highly significant

Age distribution of the patient	Asthmatic group	Nonasthmatic group	Total
	Number (%)	Number (%)	Number (%)
<25 yrs	22(22%)	33(33%)	55(27.5%)
25-30 yrs	28(28%)	43(43%)	71(35.5%)
31-35 yrs	25(25%)	14(14%)	39(19.5%)
36-40 yrs	16(16%)	9(9%)	25(12.5%)
41-45 yrs	9(9%)	1(1%)	10(5.0%)
Total	100(100%)	100(100%)	200(100.0)

Table 2 shows the distribution of the participants by gender. Among the 100 asthmatic cases, there were 21% males and 79% females; similarly, among 100 nonasthmatic cases, there were 31% males and 69% females. The difference between the asthmatic and nonasthmatic group by gender was not statistically significant (p = 0.196).

**Table 2:** Gender distribution of participants. *x*^2 ^= 3.264; p = 0.196 ns ns: not significant

Gender distribution of the participants	Asthmatic group	Nonasthmatic group	Total
	Number (%)	Number (%)	Number (%)
Male	21(21.0%)	31(31.0%)	52(26.0%)
Female	79(79.0%)	69(69.0%)	148(74.0%)
Total	100(100%)	100(100%)	200(100%)

Table 3 shows the comparison of the mean score of salivary flow rate, salivary pH, salivary buffering capacity, and salivary calcium between asthmatic and nonasthmatic groups. It was seen that the mean score of the salivary flow rate of the asthmatic group was 0.23 and that of the nonasthmatic group was 0.646. The difference was found to be significant (p < 0.001). Salivary pH value of the asthmatic group was 5.609 and that of nonasthmatic group was 6.31. A very highly significant difference was found between the mean of two groups (p < 0.001). Similarly, the mean of buffering capacity was 4.117 in the asthmatic group and 4.308 in the nonasthmatic group. Here also there was a highly significant difference between the asthmatic and nonasthmatic groups (p < 0.001). The mean salivary calcium of asthmatic group was 6.332 and that of the nonasthmatic group was 6.298 which is very close and the difference was not statistically significant (p = 0.845). The mean score of DMFT in the asthmatic group was found to be 14.05 whereas in the nonasthmatic participants it was 4.26. By applying students unpaired t-test the difference between the mean DMFT score of the asthmatic and nonasthmatic groups was found to be statistically significant (p < 0.01).

**Table 3:** Comparison of mean salivary parameters scores and DMFT scores between asthmatic group and nonasthmatic groups. unpaired  t-test *p < 0.05, statistically significant. vhs: very highly significant; ns: not significant DMFT: decayed, missing, and filled teeth

Salivary parameters and DMFT	Group	n	Mean	Std. deviation	t
Flow rate	Asthmatic	100	0.230	0.060	28.783 <0.001 vhs
	Nonasthmatic	100	0.646	0.131	
pH	Asthmatic	100	5.609	0.084	14.838 <0.001 vhs
	Nonasthmatic	100	6.310	0.465	
Buffering capacity	Asthmatic	100	4.117	0.170	8.665 <0.001 vhs
	Nonasthmatic	100	4.308	0.139	
Calcium	Asthmatic	100	6.332	1.190	0.196 p = 0.845 ns
	Nonasthmatic	100	6.298	1.209	
DMFT	Asthmatic	100	14.050	2.630	27.530 p < 0.01 vhs
	Nonasthmatic	100	4.260	2.394	

Table 4 shows the comparison of the mean number of teeth with gingival bleeding among asthmatic and nonasthmatic groups. Among the 100 nonasthmatic participants, the mean number of teeth with no gingival bleeding was 13.560 (±9.851) and among the 100 asthmatic participants, the mean number of teeth with no gingival bleeding was 9.400 (±8.095) and this difference was found to be statistically highly significant (p = 0.003) by applying Mann-Whitney U test. Likewise among the 100 asthmatic participants, the mean number of teeth with gingival bleeding was 20.700 (±8.274) which is more than the mean number of teeth with gingival bleeding found in the nonasthmatic group 16.290 (±10.418) which shows a statistically significant difference (p = 0.004). Among the 100 asthmatic participants, the mean number of teeth absent for recording the gingival bleeding was 1.780 (±2.321) and among the 100 nonasthmatic groups the mean number of teeth absent for recording the gingival bleeding was 1.830 (±1.815) and this difference was not found to be statistically significant (p = 0.254).

**Table 4:** Comparison of mean number of teeth present with gingival bleeding among asthmatic and nonasthmatic groups. *p < 0.05, statistically significant hs: highly significant; sig: significant; ns: not significant

Gingival bleeding	Group	n	Mean	Std. deviation	Z	P- value
Absence of gingival bleeding	Asthmatic	100	9.400	8.095	3.005	p = 0.003 hs
	Nonasthmatic	100	13.560	9.851		
Presence of gingival bleeding	Asthmatic	100	20.700	8.274	2.919	p = 0.004 hs
	Nonasthmatic	100	16.290	10.418		
Tooth excluded	Asthmatic	100	0.100	0.577	2.015	p = 0.044 sig
	Nonasthmatic	100	0.000	0.000		
Tooth not present	Asthmatic	100	1.780	2.321	1.140	p = 0.254 ns
	Nonasthmatic	100	1.830	1.815		

In the present study among the nonasthmatic subjects 6% of the subjects had questionable fluorosis and 3% of the subjects had very mild enamel fluorosis condition. The comparison of enamel fluorosis between asthmatic and nonasthmatic groups was found to be statistically significant (p = 0.009). Also in the present study, 8% in asthmatic group had four teeth affected by dental erosion and 2% in nonasthmatic group had five teeth affected by dental erosion. This difference between the asthmatic and nonasthmatic group was also found to be statistically significant (p = 0.003). In the asthmatic group 4% of the subjects had more than one teeth affected by dental trauma. By applying Fishers exact test a statistically significant difference was found between the asthmatic and nonasthmatic groups (p = 0.043).

 In the present study among 100 asthmatic participants 39% of the participants had score-1 (severity of loss of attachment 4-5 mm) whereas among 100 nonasthmatic participants 10% of the participants had score-1 in index teeth 31. The difference between the asthmatic and nonasthmatic group was highly significant (p < 0.001). Among the asthmatic participants 22% of the participants had score-1 and among the nonasthmatic participants 11% of the participants had score-1 in index teeth 36. The difference between the asthmatic and nonasthmatic group was statistically significant (p = 0.009). Among the asthmatic participants 16% of the participants had score-1 and among nonasthmatic participants 5% of the participants had score-1 in index teeth 46. The difference between the asthmatic and nonasthmatic group was statistically significant (p = 0.007). Among the asthmatic participants 12% of the participants had score-1 and among the nonasthmatic participants 2% of the participants had score-1 in index teeth 16 and the difference between the asthmatic and nonasthmatic group was statistically significant (p = 0.01).

In the present study, among both asthmatic and nonasthmatic participants there was no statistically significant difference in the presence of periodontal pockets.

## Discussion

The present study was a cross-sectional study conducted from May 2018 to October 2018 and was designed to evaluate salivary parameters and oral health status among asthmatic and nonasthmatic adult patients visiting the Department of Pulmonary Medicine, Yenepoya Medical College Hospital, Mangalore.

In the present study, the study group composed of 200 participants -- 100 asthmatic participants and 100 nonasthmatic participants between the age group of 18 and 45 years. The mean age of the asthmatic patients was 30 (±5.0). In a study conducted by Boskabady et al. the mean age of the asthmatic participants was 27.48 (±2.90 years) and that of nonasthmatic controls was 26.65 (±2.76 years). In the present study, the distribution of the participants by gender showed that 79% of asthmatic participants and 69% of nonasthmatic were females [10]. Ghapanchi et al. conducted a study which showed that 55% of asthmatic participants and 56% of nonasthmatic were females [11].

In the present study, a decreased mean score of salivary flow rate, pH, and buffering capacity had been found in the asthmatic group compared to that of the nonasthmatic group. Bozejac et al, where the mean value of salivary flow rate in asthmatic group 0.279 was significantly lower than in the nonasthmatic group 0.496 [12]. Lumikarim et al. in his study did not show any difference in the mean value of pH among the asthmatic group which was 7.9 and among the nonasthmatic group it was 8.1 [13].

In the present study, the nonasthmatic participants had increased enamel fluorosis compared to asthmatic participants. Also, the asthmatic participants represented a higher degree of dental erosion compared to nonasthmatic participants. Jain et al. also revealed that prevalence of dental erosion was higher in the asthmatic group (76.5%) than in the nonasthmatic group (66.7%) which showed no statistically significant difference (p = 0.272) [14].

In the present study the mean number of teeth with gingival bleeding in the asthmatic group was 20.700 which was more than the mean number teeth with gingival bleeding in the nonasthmatic group (16.290) which shows a statistically significant difference. It is similar to the study finding by Gomes-Filho et al. where the mean value of gingival bleeding in the asthmatic group (28.43) was more than the mean value found in the nonasthmatic group (14.69) which shows a statistically significant difference [15].

In the present study, the loss of attachment score in index teeth 16, 31, 36, and 46 was higher in the asthmatic group compared to that of nonasthmatic groups. Bhardwaj also showed similar findings where the mean score loss of attachment in the asthmatic group was higher than the mean score loss of attachment in the nonasthmatic group with statistically significant difference (p < 0.004) [16].

In the present study, the mean number of teeth with periodontal pocket in the asthmatic group did not show any consistent difference with the mean number of teeth with periodontal pocket in a nonasthmatic group. Shulman et al. also showed that mean number of teeth with periodontal pocket in the asthmatic group was 2.0 and did not show any consistent difference with the mean number of teeth with periodontal pocket in the nonasthmatic group (2.1) [17].

 The present study also showed no significant oral mucosal lesion findings in the asthmatic group compared to the nonasthmatic group. Ghapanchi et al. showed a different finding in which 13(13%) of asthmatic participants had chronic atrophic candidiasis, 13(13%) of them had fissured tongue, 10(10%) of them had geographic tongue and in the control group 2(2%) of nonasthmatic participants had geographic tongue, 11(11%) of them had fissured tongue, and 2(2%) of them had lichenoid reaction [11].

The potential limitation of the present study was that the present findings involved only patients visiting Yenepoya Medical College Hospital and may underestimate the real burden as the present study is bound to a particular tertiary care hospital and not the entire population of Mangalore city.

## Conclusions

The results from the present study support the hypothesis that asthmatic participants have a higher risk of oral diseases than nonasthmatic subjects. In the present study, it was found that asthmatic patients face an increased risk of dental caries due to low salivary flow rate and pH buffering capacity which is due to inhaler therapy. Also, the present study reveals a positive association between asthma and periodontal disease. Asthmatic participants are at increased risk of dental erosion and dental trauma compared to nonasthmatic participants. There was no risk of dental fluorosis in asthmatic participants compared to nonasthmatic participants. Also in the present study, no sign of oral mucosal lesions was found in the participants of both groups. Hence in order to prevent disease progression in the hard dental tissue and oral cavity of asthmatic patients, it is necessary to apply adequate preventive measures and motivate their continuous application in this caries-risk population.
